# Molecular identification and microbiome profiling of household casebearer, *Phereoeca* sp. (Lepidoptera: Tineidae) from Malaysia: Potential implications for human skin irritation

**DOI:** 10.1371/journal.pone.0346590

**Published:** 2026-04-09

**Authors:** Salmah Yaakop, Muhammad Afiq Senen, Nur Alya Adila Rosli, Muhamad Azmi Mohammed

**Affiliations:** 1 Centre for Insect Systematics, Faculty of Science and Technology, Universiti Kebangsaan Malaysia, Bangi, Selangor, Malaysia; 2 Department of Crop Science, Faculty of Agricultural and Forestry Sciences, Universiti Putra Malaysia Sarawak, Bintulu, Sarawak, Malaysia; Universiti Teknologi Malaysia - Main Campus Skudai: Universiti Teknologi Malaysia, MALAYSIA

## Abstract

In Malaysia, anecdotal accounts have linked the household casebearer (Lepidoptera: Tineidae) to skin lesions and localized inflammation; however, scientific evidence is lacking, and the species’ taxonomic identity remains unclear. This study aimed to confirm the species identity and examine the bacteria associated with larvae that may be linked to skin irritation. Larvae were collected from three locations in Peninsular Malaysia and preserved. DNA was extracted from the larvae, and species identification was conducted by analyzing the cytochrome c oxidase subunit I (COI) gene through DNA barcoding. To study the bacteria present, the bacterial 16S rRNA gene was amplified and sequenced using Next-generation sequencing technology. The DNA sequences were analyzed to determine the species and profile the bacterial communities. The results identified the specimens as *Phereoeca* sp., suggesting they may represent an undescribed lineage. Microbiome analysis revealed that Proteobacteria (40.18%) and Actinobacteriota (32.13%) were the dominant bacterial phyla, with *Cutibacterium acnes*, *Enterobacter*, and *Pseudomonas* among the taxa previously associated with skin irritation or opportunistic infections. Several unclassified but potentially relevant taxa were also identified. These findings provide new insights into the microbial ecology and taxonomy of *Phereoeca* and underscore its potential role in medically significant interactions within human environments.

## Introduction

Casebearers (Lepidoptera: Tineidae) are small moths commonly encountered in domestic environments and known for their larval habit of constructing silk cases adorned with fibers, debris, and other environmental materials [1]. These insects, often referred to as “plaster bagworms” or “household casebearers,” or locally known as “kamitetep,” are widespread in Malaysia and are frequently observed on walls, ceilings, and household fabrics. While their presence is mostly viewed as a nuisance due to fabric damage, there is growing concern over their potential role in causing allergic skin reactions.

Social media reports and anecdotal accounts have linked contact with these larvae to skin irritation and inflammatory responses in humans. Despite these claims, no scientific studies have confirmed the dermatological effects or identified the specific species involved. Moreover, the taxonomy of *Phereoeca* species in Southeast Asia remains poorly resolved, with limited molecular data available for accurate identification.

Insects, including moths, harbor diverse microbiomes that play key roles in host biology, digestion, immunity, and environmental interactions [Zhang et al., 2022]. Microbial communities may include bacterial taxa capable of producing bioactive compounds such as histamine, a biogenic amine implicated in immune regulation, allergic reactions, and inflammation [2]. Several bacterial genera, including *Pseudomonas*, possess the enzymatic capacity to convert L-histidine into histamine through histidine decarboxylase activity [3]. These associations suggest that the microbiota of household insects may be medically relevant.

Recent advances in Next-generation sequencing (NGS) have enabled comprehensive profiling of microbial communities associated with insects through metagenomic approaches [4]. Metagenomic sequencing allows the identification of both culturable and unculturable microbes, offering insights into host–microbe interactions and potential health implications [5,6]. However, no studies to date have applied these methods to the casebearer in Malaysia, leaving a gap in our understanding of its microbiome and possible effects on human health.

This study aims to address these gaps by combining DNA barcoding and 16S rRNA-based microbiome profiling of *Phereoeca* sp. collected from Malaysian households. The objectives are to (i) confirm species identity using the COI gene, (ii) characterize the associated bacterial community, and (iii) identify microbial taxa potentially involved in skin irritation. The findings are expected to provide both taxonomic clarity and novel insights into the possible medical relevance of this common household insect.

## Materials and methods

**Fig 1:**
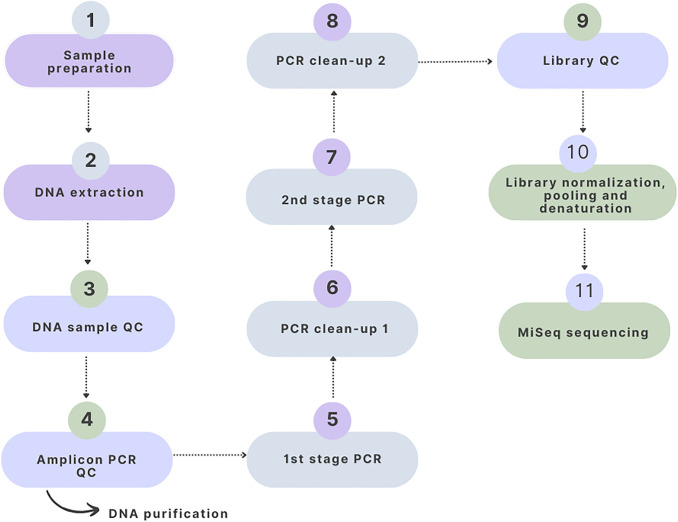
Workflow of DNA extraction, PCR amplification, and Next-generation sequencing used for microbiome profiling of *Phereoeca* sp. larvae. The process includes sample preparation, DNA extraction, DNA quality control, amplicon PCR, two-stage PCR amplification, PCR clean-up steps, library quality control, library normalization and pooling, followed by Illumina MiSeq sequencing.

The overall experimental workflow used for molecular identification and microbiome analysis of *Phereoeca* sp. larvae is summarized in Fig 1. The procedure included sample preparation, DNA extraction, PCR amplification, library preparation, and Illumina MiSeq sequencing, followed by bioinformatic analysis.

### Sampling location and household casebearer collection

Sampling was conducted at three locations in the western part of Peninsular Malaysia: Bangi (Selangor), Benut (Johor), and Nilai (Negeri Sembilan). These sites were selected to provide an overview of microbial composition. Visual observations were carried out in indoor residential environments by examining the surfaces of ceilings and walls with the naked eye. The larval stage of the moth, found within portable cases, was collected and preserved in 70% alcohol. Some larvae were reared in a plastic container (10 × 8 × 6 cm; dry sample) until adult emergence. Larval sampling was conducted within private residential premises with homeowner consent. The study did not involve protected species or protected areas; therefore, no specific collection permit was required for this study. Representative voucher specimens of *Phereoeca* sp. from this study were deposited in the Centre for Insect Systematics, Universiti Kebangsaan Malaysia (UKM) collection to support future taxonomic work.

### Morphological identification

**Fig 2:**
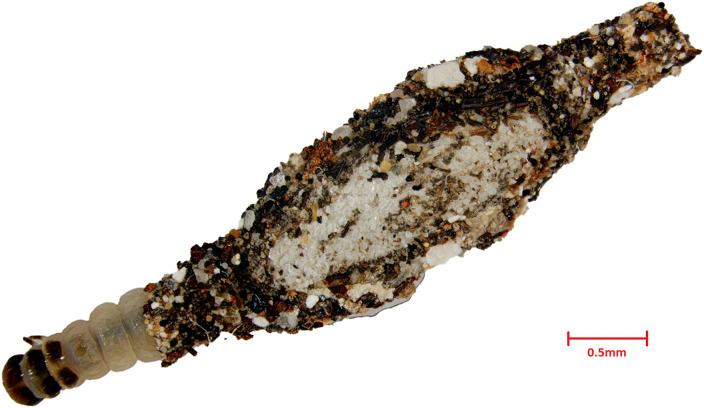
Representative larva of the household casebearer *Phereoeca* sp. collected from indoor environments in Peninsular Malaysia. The larva is enclosed within a portable case constructed from silk, dust particles, and environmental debris. Scale bar = 0.5 mm.

Each individual larval sample was recorded and photographed using an Image Analyser (Zeiss Stereo Discovery V20) with AxioVision software, and a representative image is presented in Fig 2. Only samples with similar structures were assumed to belong to the same species. Selected individuals that were successfully reared to the adult stage were identified to species level using a stereo microscope (Zeiss, Germany) at 40× magnification. To further support the identification, DNA barcode analysis was conducted using a minimum of three samples from each location.

### DNA Barcoding Analysis

#### DNA extraction and PCR amplification.

DNA was extracted from nine larval samples of the household casebearer (Table 1). To minimize external contamination, all specimens were handled using sterile forceps and transferred into sterile containers. Prior to DNA extraction, larvae were carefully removed from their cases and rinsed three times with 70% ethanol followed by sterile distilled water to reduce surface-associated contaminants. Microbiome analysis was performed on larval material only, and the external casing was not included in downstream DNA extraction unless otherwise stated. All dissections were conducted under clean laboratory conditions. Only the larval portion was collected for extraction, which was performed using the Macherey-Nagel DNA Kit (Germany) according to the manufacturer’s protocols.

**Table 1:** List of *Phereoeca* sp. larval samples collected from three localities (Nilai, Benut, Bangi), including sample codes, species identification, and type of molecular analysis performed (*COI* barcoding or 16S rRNA metagenomics).

No.	Sample code	Species	Locality	Analysis (gene)
1	1A	*Phereoeca* sp.	Nilai, Negeri Sembilan, Malaysia	Barcode (*COI*)
2	2A	*Phereoeca* sp.	Nilai, Negeri Sembilan, Malaysia	Barcode (*COI*)
3	3A	*Phereoeca* sp.	Nilai, Negeri Sembilan, Malaysia	Barcode (*COI*)
4	1B	*Phereoeca* sp.	Benut, Johor, Malaysia	Barcode (*COI*)
5	2B	*Phereoeca* sp.	Benut, Johor, Malaysia	Barcode (*COI*)
6	3B	*Phereoeca* sp.	Benut, Johor, Malaysia	Barcode (*COI*)
7	1C	*Phereoeca* sp.	Bangi, Selangor, Malaysia	Barcode (*COI*)
8	2C	*Phereoeca* sp.	Bangi, Selangor, Malaysia	Barcode (*COI*)
9	3C	*Phereoeca* sp.	Bangi, Selangor, Malaysia	Barcode (*COI)*
10	1A	*Phereoeca* sp.	Nilai, Negeri Sembilan, Malaysia	Metagenome (*16S*)
11	2A	*Phereoeca* sp.	Nilai, Negeri Sembilan, Malaysia	Metagenome (*16S*)
12	3A	*Phereoeca* sp.	Nilai, Negeri Sembilan, Malaysia	Metagenome (*16S*)
13	1B	*Phereoeca* sp.	Benut, Johor, Malaysia	Metagenome (*16S*)
14	2B	*Phereoeca* sp.	Benut, Johor, Malaysia	Metagenome (*16S*)
15	3B	*Phereoeca* sp.	Benut, Johor, Malaysia	Metagenome (*16S*)
16	1C	*Phereoeca* sp.	Bangi, Selangor, Malaysia	Metagenome (*16S*)
17	2C	*Phereoeca* sp.	Bangi, Selangor, Malaysia	Metagenome (*16S*)
18	3C	*Phereoeca* sp.	Bangi, Selangor, Malaysia	Metagenome (*16S*)

PCR analysis targeted the cytochrome c oxidase subunit I (*COI*) gene using primer pairs from Folmer et al. [7]: LCO1490 (5′-GGTCAACAAATCATAAAGATATTGG-3′) and HCO2198 (5′-TAAACTTCAGGGTGACCAAAAAATCA-3′). Each 25 μL PCR reaction contained 12.5 μL Mastermix (Promega), 5.5 μL ddH₂O, and 5 μL of DNA template (4–6 μL). PCR conditions followed Mohammed et al. [8], Yaakop et al. [9], and Mohammed et al. [10]. PCR products were subjected to electrophoresis for 30 min at 90 V using a 1.5% agarose gel.

#### DNA sequencing, editing, and BLAST analyses.

**Fig 3:**
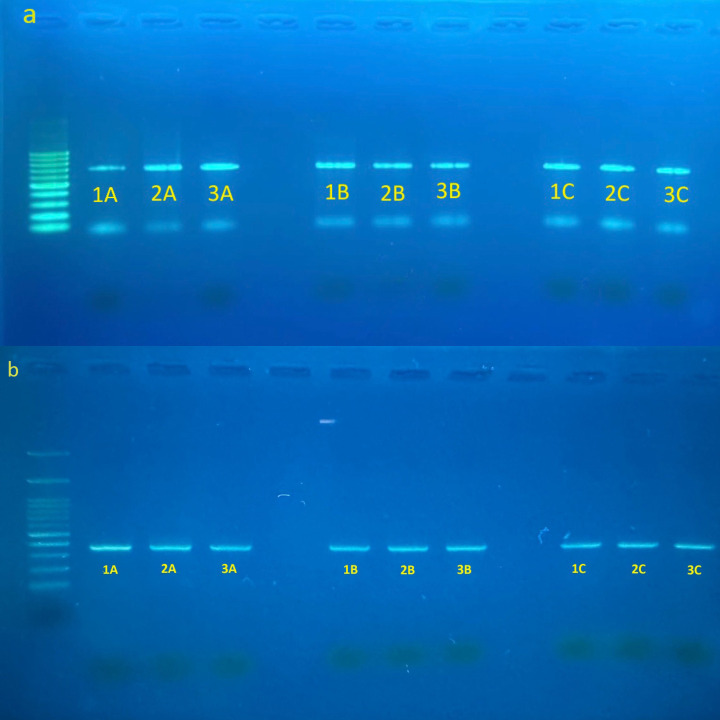
Agarose gel electrophoresis of PCR amplification products obtained from *Phereoeca* sp. larval samples. (a) Amplification of the mitochondrial cytochrome c oxidase subunit I (COI) gene used for DNA barcoding. (b) Amplification of the bacterial 16S rRNA gene (V3-V4 region) used for microbiome analysis. Sample codes correspond to specimens collected from three localities: Nilai (1A-3A), Benut (1B–3B), and Bangi (1C-3C).

PCR amplicons were purified and sequenced commercially by Apical Scientific Sdn. Bhd. (Malaysia). Raw sequence chromatograms were inspected and edited using *Sequencher* version 5.4.6 (Gene Codes Corporation, USA). Consensus sequences were subsequently compared against reference sequences in the National Center for Biotechnology Information (NCBI) database using the Basic Local Alignment Search Tool (BLAST) [11] to verify species identity. Successful amplification of the mitochondrial cytochrome *c* oxidase subunit I (COI) gene was confirmed through agarose gel electrophoresis (Fig 3a). DNA barcoding analyses were conducted on nine specimens collected from three localities in Malaysia (Table 2). The validated COI sequences were deposited in GenBank under accession numbers PP961802–PP961804, PQ882320, and PQ885044-PQ885048.

**Table 2:** COI sequences used in phylogenetic analysis of *Phereoeca* spp., including reference sequences retrieved from GenBank and sequences generated in the present study.

No.	Voucher/ Sample ID	Species designation	Locality	GenBank accession no.	Source
1	BR3	*Metisa plana*	Malaysia	MK548641	GenBank
2	BR4	*Metisa plana*	Malaysia	MK548642	GenBank
3	USNM ENT 00662028	*Phereoeca uterella*	Papua New Guinea	GU695649	GenBank
4	Pha1	*Phereoeca uterella*	India	OQ740257	GenBank
5	*P. uterella*	*Phereoeca uterella*	China	HM016801	GenBank
6	*P. praecox*	*Phereoeca praecox*	USA (South Carolina)	KY575118	GenBank
7	UFV1	*Phereoeca* sp.	Brazil	MH540351	GenBank
8	Lep23	*Phereoeca* sp.	Malta	MW305963	GenBank
9	DNAYLL18052	*Pelecystola peculiaris*	China	MW396737	GenBank
10	DNAYLL18053	*Pelecystola strigosa*	China	MW396739	GenBank
11	1A	*Phereoeca* sp. (Malaysia)	Negeri Sembilan, Malaysia	PP961802	This study
12	2A	*Phereoeca* sp. (Malaysia)	Negeri Sembilan, Malaysia	PQ882320	This study
13	3A	*Phereoeca* sp. (Malaysia)	Negeri Sembilan, Malaysia	PQ885044	This study
14	1B	*Phereoeca* sp. (Malaysia)	Johor, Malaysia	PP961803	This study
15	2B	*Phereoeca* sp. (Malaysia)	Johor, Malaysia	PQ885045	This study
16	3B	*Phereoeca* sp. (Malaysia)	Johor, Malaysia	PQ885046	This study
17	1C	*Phereoeca* sp. (Malaysia)	Selangor, Malaysia	PP961804	This study
18	2C	*Phereoeca* sp. (Malaysia)	Selangor, Malaysia	PQ885047	This study
19	3C	*Phereoeca* sp. (Malaysia)	Selangor, Malaysia	PQ885048	This study

#### Tree reconstruction.

Sequences were analyzed using the Neighbor-Joining (NJ) method based on Kimura 2-Parameter (K2P) distances in PAUP 4. Bootstrap analysis was performed with 1,000 replications. Maximum Parsimony (MP) analysis was also conducted using heuristic search with TBR and 1,000 bootstrap replicates. Additional sequences from expected species in GenBank were included (Table 2).

### Metagenomic analysis

#### Larvae extraction.

Nine larvae collected from three locations (Table 1) were dissected from their cases with sterile forceps. Samples were rinsed three times with 70% alcohol. DNA was extracted using the Macherey-Nagel DNA Kit. Samples <40 mg were placed in NucleoSpin® Bead Tubes with buffers BE, MG, and proteinase K. Samples were vortexed, centrifuged, washed with BW and B5 buffers, dried, and eluted with BE buffer.

#### DNA quality control.

DNA purity and concentration were assessed using the Implen NanoPhotometer® N60/N50 and the iQuant™ Broad Range dsDNA Quantification Kit. Only high-quality DNA proceeded to downstream applications.

#### Amplicon PCR of bacterial 16S rRNA.

DNA extracted from nine *Phereoeca* sp. larval specimens was subjected to amplification prior to bacterial 16S rRNA gene metabarcoding analysis. The V3-V4 hypervariable regions of the bacterial 16S rRNA gene were amplified using primers Bakt_341F and Bakt_805R [12]. PCR reactions were performed using REDiant 2 × PCR Master Mix (1st BASE, Malaysia) following the manufacturer’s recommendations. Amplification products were verified by agarose gel electrophoresis before downstream metabarcode sequencing analysis (Fig 3b).

### Library construction

#### First PCR.

Overhang adapters were attached during amplification. Reactions were conducted using KOD-Multi & Epi DNA polymerase in 30 cycles.

#### Second PCR (Indexing).

Dual indices were added using the Illumina Nextera XT Index Kit v2. Library quality was verified using an Agilent Bioanalyzer 2100 and Helixyte Green™.

#### Next-generation sequencing.

Libraries were normalized and pooled before sequencing on the Illumina MiSeq (300 PE). Raw sequences (PRJNA897111) were processed in R v3.6.2. Using DADA2 [13], sequences were quality-filtered, denoised, spliced, and checked for chimeras. Taxonomy was assigned using RDP’s Naïve Bayesian Classifier with SILVA v138.1 [14] in QIIME2 [15].

Relative abundance was visualized using phyloseq [16]. Venn diagrams, rarefaction curves, and phylogenetic trees (FastTree2; [17]) were constructed. Sequences <150 bp or >600 bp were removed.

### Statistical analyses

Alpha diversity metrics (Chao1, Observed ASVs, Shannon, Simpson) were calculated in R v3.6.2. Samples were rarefied to the lowest depth. Differences among localities were tested using Wilcoxon rank-sum tests (p ≤ 0.05). Beta diversity was examined using Bray–Curtis and UniFrac distances. PCoA and UPGMA analyses were conducted. ANOSIM tested for community composition differences.

## Results

### DNA barcoding

Sequence similarity analysis of nine individuals collected from three localities in Malaysia revealed that the obtained sequences shared only 89.26–90.08% identity with available *Phereoeca uterella* sequences in GenBank, suggesting that the specimens do not represent the same species. Accordingly, the Malaysian material is provisionally designated as *Phereoeca* sp., pending further taxonomic confirmation.

**Fig 4:**
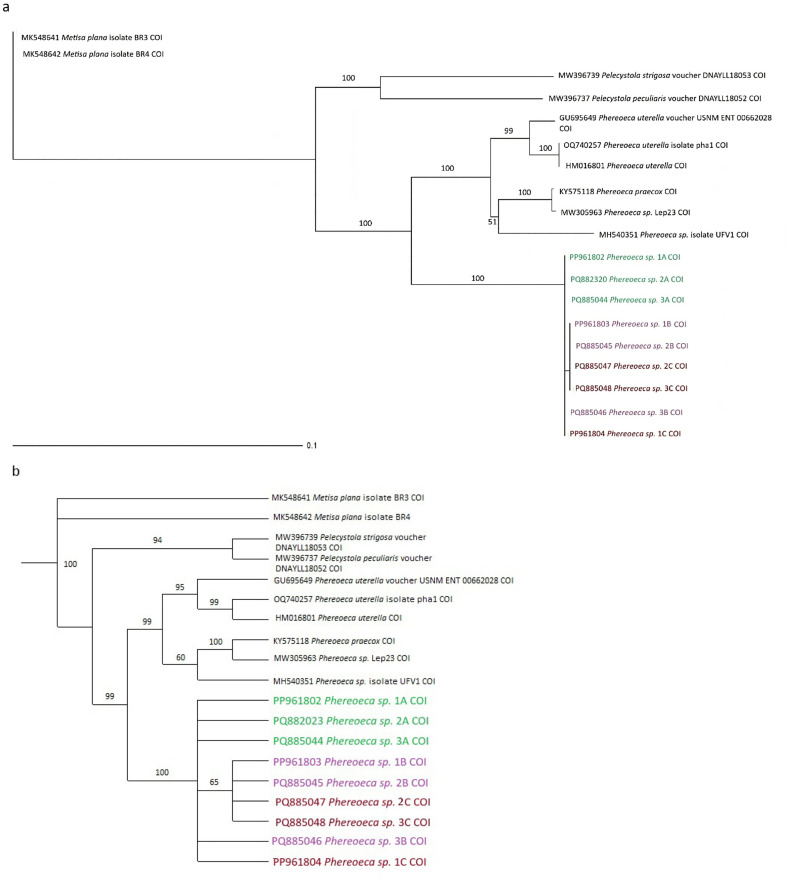
Phylogenetic relationships of *Phereoeca* sp. inferred from mitochondrial COI sequences. (a) Neighbor-Joining (NJ) tree constructed using Kimura 2-parameter genetic distances. (b) Maximum Parsimony (MP) tree reconstructed from the same dataset. Bootstrap support values are indicated at the nodes. Malaysian *Phereoeca* specimens form a distinct clade separate from reference sequences retrieved from GenBank.

This distinction is further supported by phylogenetic analyses using both Neighbor-Joining (NJ) and Maximum Parsimony (MP) methods, with the Malaysian specimens forming a separate clade from *P. uterella* individuals from other countries (Figs 4a and 4b). The NJ and MP trees provided strong statistical support for this separation, with bootstrap values of 100% and 99%, respectively. These findings suggest that the Malaysian population may represent a distinct, previously undescribed lineage within the genus *Phereoeca*. Given the significant genetic divergence and phylogenetic separation, the species is currently referred to as *Phereoeca* sp. Malaysia. Further morphological and molecular investigations are required to clarify its taxonomic status and determine whether it constitutes a novel species.

### Genetic distance

Fig 4a presents the Neighbor-Joining (NJ) tree constructed from the COI dataset, with bootstrap values indicated on the corresponding branches. The analysis revealed that genetic divergence between different genera ranged from 0.15959 to 0.19355, indicating substantial intergeneric variation (Tables 3 and 4). In contrast, intraspecific divergence between individuals of the same species was considerably lower, ranging from 0.00000 to 0.06112, consistent with expected values for conspecific populations. Notably, the *Phereoeca* sp. specimens from Malaysia exhibited a genetic divergence of 0.10357 to 0.10526 when compared with *P. uterella* sequences from other geographic regions. This level of divergence is significantly higher than what is typically observed within a single species, suggesting that the Malaysian specimens may represent a distinct taxonomic entity. These findings support the hypothesis that the *Phereoeca* population in Malaysia constitutes a potentially undescribed species, warranting further taxonomic investigation through morphological and additional molecular analyses.

**Table 3:** Pairwise genetic distance matrix of *COI* sequences between *Phereoeca* sp. and related Lepidoptera species based on Kimura 2-parameter model.

	1	2	3	4	5	6	7	8	9	10	11	12	13	14	15
1.MK548641 *Metisa plana*	–														
2.MK548642 *Metisa plana*	0.0000	–													
3.MW396739 *Pelecystola strigosa*	0.19355	0.19355	–												
4.MW396737 *Pelecystola peculiaris*	0.17487	0.17487	0.11545	–											
5.GU695649 *Phereoeca uterella*	0.18166	0.18166	0.16808	0.15959	–										
6.OQ740257 *Phereoeca uterella*	0.18336	0.18336	0.17827	0.15789	0.01868	–									
7.HM016801 *Phereoeca uterella*	0.18336	0.18336	0.17827	0.15789	0.01868	0.0000	–								
8.KY575118 *Phereoeca praecox*	0.19015	0.19015	0.17148	0.16469	0.03905	0.04414	0.04414	–							
9.MH540351 *Phereoeca* sp.	0.19185	0.19185	0.17317	0.16638	0.06621	0.06112	0.06112	0.05093	–						
	1	2	3	4	5	6	7	8	9	10	11	12	13	14	15
10.*Phereoeca* sp. 1A	0.19185	0.19185	0.16129	0.16638	0.10357	0.10357	0.10357	0.10017	0.11715	–					
11.*Phereoeca* sp. 2A	0.19185	0.19185	0.16129	0.16638	0.10357	0.10357	0.10357	0.10017	0.11715	0.0000	–				
12*.Phereoeca* sp. 3A	0.19185	0.19185	0.16129	0.16638	0.10357	0.10357	0.10357	0.10017	0.11715	0.0000	0.0000	–			
13*.Phereoeca* sp. 1B	0.19355	0.19355	0.16299	0.16808	0.10526	0.10526	0.10526	0.10187	0.11885	0.0017	0.0017	0.0017	–		
14*.Phereoeca* sp. 2B	0.19355	0.19355	0.16299	0.16808	0.10526	0.10526	0.10526	0.10187	0.11885	0.0017	0.0017	0.0017	0.0000	–	
15*.Phereoeca* sp. 3B	0.19185	0.19185	0.16129	0.16638	0.10357	0.10357	0.10357	0.10017	0.11715	0.0000	0.0000	0.0000	0.0017	0.0017	–
16*.Phereoeca* sp. 1C	0.19185	0.19185	0.16129	0.16638	0.10357	0.10357	0.10357	0.10017	0.11715	0.0000	0.0000	0.0000	0.0017	0.0017	0.0000
17.*Phereoeca* sp. 2C	0.19355	0.19355	0.16299	0.16808	0.10526	0.10526	0.10526	0.10187	0.11885	0.0017	0.0017	0.0017	0.0000	0.0000	0.0017
18.*Phereoeca* sp. 3C	0.19355	0.19355	0.16299	0.16808	0.10526	0.10526	0.10526	0.10187	0.11885	0.0017	0.0017	0.0017	0.0000	0.0000	0.0017
19.MW305963 *Phereoeca* sp.	0.18846	0.18846	0.16978	0.16299	0.03735	0.04584	0.04584	0.0017	0.05263	0.10187	0.10187	0.10187	0.10357	0.10357	0.10187

**Table 4:** Continuation of Table 4 showing additional genetic distance values among *Phereoeca* sp. specimens from different locations and reference sequences.

	16	17	18	19
16*.Phereoeca* sp. 1C	–			
17.*Phereoeca* sp. 2C	0.0017	–		
18.*Phereoeca* sp. 3C	0.0017	0.0000	–	
19.MW305963 *Phereoeca* sp.	0.10187	0.10357	0.10357	–

### Bacterial community diversity

**Fig 5:**
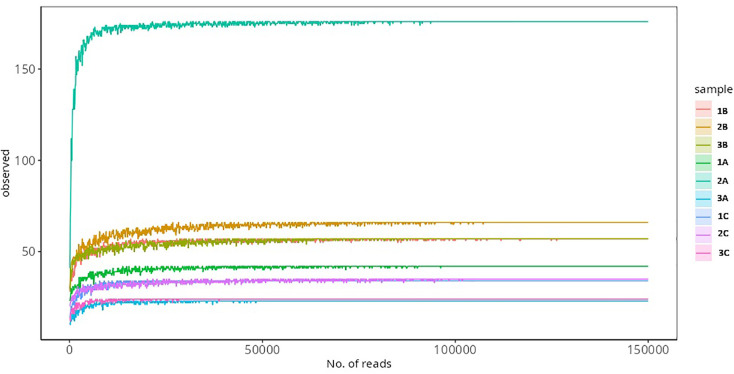
Rarefaction curves showing sequencing depth and bacterial diversity recovered from *Phereoeca* sp. larvae. The curves represent the number of observed amplicon sequence variants (ASVs) as a function of sequencing reads across samples from Johor, Negeri Sembilan (Nilai), and Selangor (Bangi). Curves approaching a plateau indicate adequate sequencing coverage.

Sequencing of the V3-V4 hypervariable regions of the bacterial 16S rRNA gene yielded a total of 996,762 raw reads, with an average of 110,751 reads per sample. Rarefaction curves for all three biological replicates approached asymptotes, indicating sufficient sequencing depth and sampling effort to capture the majority of bacterial diversity across all localities (Fig 5). Following quality filtering and removal of low-quality and short reads, final read counts were 163,137 for Johor (J), 142,090 for Negeri Sembilan (S), and 123,284 for Selangor (U) samples (Table 1). A total of 461 Amplicon Sequence Variants (ASVs) were identified and used for clustering and downstream taxonomic classification.

**Fig 6:**
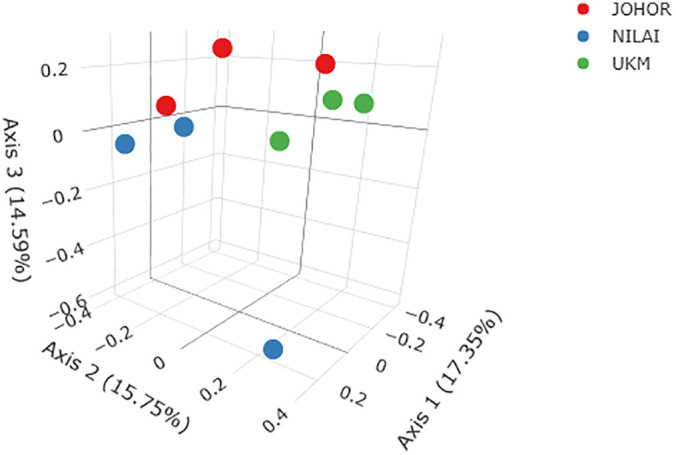
Principal Coordinate Analysis (PCoA) plot illustrating differences in bacterial community composition associated with *Phereoeca* sp. larvae collected from three locations: Johor, Nilai (Negeri Sembilan), and Bangi (Selangor). Samples cluster according to similarities in microbial community structure.

**Fig 7:**
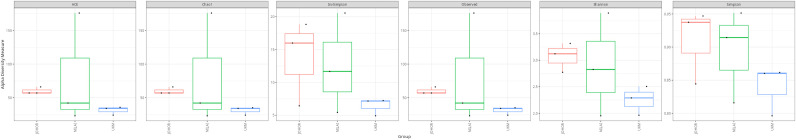
Comparison of alpha diversity indices for bacterial communities associated with *Phereoeca* sp. larvae across sampling localities. Diversity metrics include ACE, Chao1, Inverse Simpson, Observed ASVs, Shannon index, and Simpson index.

Alpha diversity metrics, including observed ASVs, Chao1 richness estimator, Shannon index, and Simpson index, revealed variation in microbial diversity across samples. The sample from Negeri Sembilan (S2) exhibited the highest alpha diversity (observed ASVs = 176; Shannon index = 3.89; Simpson index = 0.95), while Selangor (U3) showed the lowest diversity (observed ASVs = 24). Comparatively, the median Shannon index across locations was highest in Johor (3.12), followed by Negeri Sembilan (2.83), and lowest in Selangor (2.29) (Table 5). Statistically significant differences in bacterial community diversity across localities were evident in all diversity indices, as visualized in Figs 6 and 7.

**Table 5:** Summary of sequencing output and microbial alpha diversity indices (Shannon, Simpson, Chao1, Observed ASVs) for *Phereoeca* sp. samples from Johor, Negeri Sembilan, and Selangor, Malaysia.

Locality	Code	Sequence number		Alpha diversity			
		No. of raw reads	No. of reads after qualify filtering	Shannon index (H’)	Simpson index	Chao1 index	Observed index
Johor, Malaysia	J1	142,520	191,067	3.12	0.94	57.00	57.00
	J2	126,045	170,151	3.32	0.95	66.00	66.00
	J3	96,690	128,193	2.77	0.84	57.00	57.00
	Mean ± SD	121,752 ± 23,215	163,137 ± 32,018	3.07 ± 0.28	0.91 ± 0.06	60.00 ± 5.20	60.00 ± 5.20
N. Sembilan, Malaysia	S1	116,860	152,020	2.83	0.91	42.00	42.00
	S2	103,441	156,775	3.89	0.95	176.00	176.00
	S3	98,037	117,476	1.95	0.82	23.00	23.00
	Mean ± SD	106,113 ± 9,692	142,090 ± 21,449	2.89 ± 0.97	0.89 ± 0.07	80.33 ± 83.39	80.33 ± 83.39
Selangor, Malaysia	U1	92,053	111,519	2.29	0.86	34.00	34.00
	U2	107,445	132,792	2.51	0.86	35.00	35.00
	U3	113,671	125,542	1.96	0.80	24.00	24.00
	Mean ± SD	104,390 ± 11,128	123,284 ± 10,815	2.25 ± 0.28	0.84 ± 0.03	31.00 ± 6.08	31.00 ± 6.08

**Fig 8:**
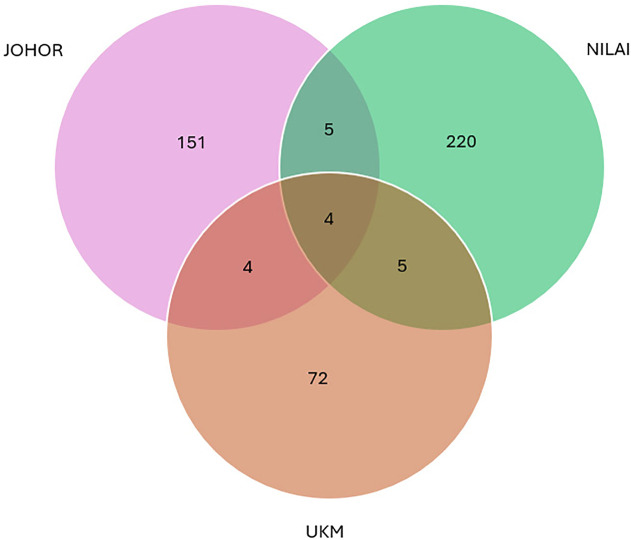
Venn diagram showing the number of shared and unique amplicon sequence variants (ASVs) among *Phereoeca* sp. samples from Johor, Nilai (Negeri Sembilan), and Bangi (Selangor).

**Fig 9:**
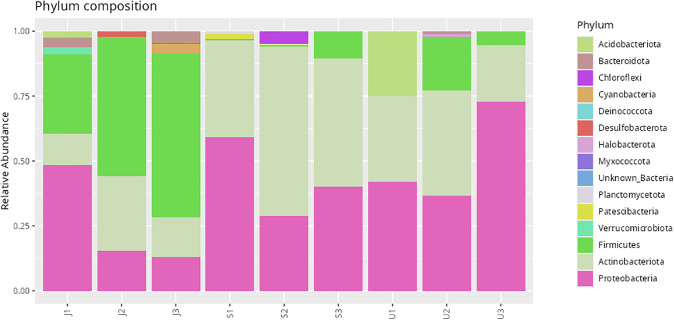
Relative abundance of bacterial phyla detected in *Phereoeca* sp. larvae from different sampling locations. Each bar represents an individual sample, and colors indicate the proportion of major bacterial phyla identified through 16S rRNA gene sequencing.

**Fig 10:**
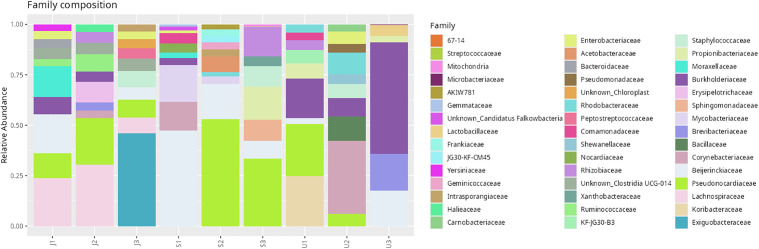
Relative abundance of dominant bacterial families associated with *Phereoeca* sp. larvae across sampling localities. Each bar represents the microbial composition of individual samples.

**Fig 11:**
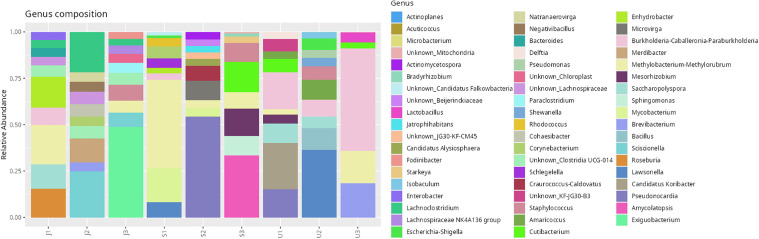
Relative abundance of bacterial genera detected in the microbiome of *Phereoeca* sp. larvae. Bars represent individual samples, showing the distribution of dominant genera across different sampling sites.

Among the 461 ASVs, four bacterial taxa were shared across all three populations, namely *Paraburkholderia* (Proteobacteria), *Methylobacterium* (Alphaproteobacteria), and *Cutibacterium* (Actinobacteria). The highest ASV richness was recorded in samples from Nilai (S, 151 ASVs), followed by Johor (J, 123 ASVs), and Selangor (U, 72 ASVs) (Fig 8). Taxonomic classification revealed that the dominant phylum was Proteobacteria (40.18%), followed by Actinobacteriota (32.73%) and Firmicutes (21.22%) (Fig 9). At the class level, Actinobacteria (31.77%), Alphaproteobacteria (21.76%), and Gammaproteobacteria (18.42%) were most abundant, with other classes distributed among remaining phyla (Table 6). At the family level, *Pseudonocardiaceae* (15.53%), *Beijerinckiaceae* (12.97%), and *Burkholderiaceae* (11.24%) were the most prevalent (Fig 10). Although *Enterobacteriaceae* was present, its relative abundance was low (1.7%). Genus-level analysis identified *Methylobacterium* and *Methylorubrum* (combined 11.4%), *Burkholderia-Caballeronia-Paraburkholderia* (10.7%), and *Pseudonocardia* (5.7%) among the most dominant genera (Fig 11). The genus *Enterobacter*, of particular interest in this study, was detected at a relative abundance of 0.71%.

**Table 6:** Top 25 most abundant bacterial classes identified in *Phereoeca* sp. samples across three locations, based on relative abundance.

Class	Percentage relative abundance (%)
Actinobacteria	31.77
Alphaproteobacteria	21.76
Gammaproteobacteria	18.42
Clostridia	11.37
Bacilli	9.85
Acidobacteriae	2.30
Bacteroidia	1.01
Chloroflexia	0.50
Verrucomicrobiae	0.40
Cyanobacteriia	0.38
Subgroup 5	0.36
Coriobacteriia	0.34
Acidimicrobiia	0.30
Desulfovibrionia	0.27
ABY1	0.22
Thermoleophilia	0.21
Planctomycetes	0.15
Halobacteria	0.11
Rubrobacteria	0.10
Ignavibacteria	0.08
Saccharimonadia	0.03
Unknown_Bacteria	0.02
Deinococci	0.02
**Class**	**Percentage relative abundance (%)**
Ktedonobacteria	0.01
Parcubacteria	0.01
Polyangia	0.00
Holophagae	0.00

At the species level, *Cutibacterium acnes* (3.11%) emerged as one of the top 10 most abundant species and was among the top seven families. Other notably abundant species included *Enhydrobacter aerosaccus* and *Mesorhizobium loti*, ranked 14th and 15th among the top 25 most abundant taxa, respectively (Table 7). Several unidentified or unclassified species were also present and are discussed in the context of their potential ecological or medical relevance. Taxonomic assignments were conducted using the SILVA reference database, which revealed a total of 15 bacterial phyla, including one unclassified group. Taxonomic composition across samples was visualized through taxa boxplots, which consistently showed Proteobacteria as the most dominant phylum across all *Phereoeca* specimens.

**Table 7:** Top 25 most abundant bacterial species (classified and unclassified) detected in *Phereoeca* sp. larvae, with corresponding relative abundance percentages.

Species	Percentage relative abundance (%)
Unknown *Methylobacterium-Methylorubrum*	11.79
Unknown *Burkholderia-Caballeronia-Paraburkholderia*	10.99
Unknown *Pseudonocardia*	5.70
Unknown *Lawsonella*	4.51
Unknown *Exiguobacterium*	3.72
Unknown *Saccharopolyspora*	3.33
Unknown *Amycolatopsis*	3.27
*Cutibacterium acnes* (*C. acnes*)	2.95
Unknown *Staphylococcus*	2.65
Unknown *Sciscionella*	2.64
Unknown *Lachnoclostridium*	2.61
Unknown Brevibacterium	2.54
Unknown Candidatus Koribacter	2.29
*Enhydrobacter aerosaccus (E. aerosaccus)*	2.16
*Mesorhizobium loti (M. loti)*	1.88
Unknown *Amaricoccus*	1.83
**Species**	**Percentage relative abundance (%)**
Unknown *Clostridia* UCG-014	1.76
Unknown *Roseburia*	1.70
Unknown *Mycobacterium*	1.47
Unknown *Bacillus*	1.21
Unknown *Merdibacter*	1.19
Unknown *Corynebacterium*	1.15
Unknown Lachnospiraceae	1.06

## Discussion

### Taxonomic uncertainty of *Phereoeca* sp. in Malaysia

The household casebearer, *Phereoeca* sp., remains underrepresented in entomological research, both in Malaysia and globally, despite its common presence in household environments (Villanueva- Jimenez and Fasulo 2010). Public attention in Malaysia was recently sparked by viral social media posts alleging that this insect causes skin irritation or bites, although no empirical data have validated such claims. The species status of Malaysian *Phereoeca* specimens remains unclear due to slight morphological differences compared to the globally accepted *P. uterella*. DNA barcoding in this study revealed that Malaysian samples did not cluster with known *Phereoeca* species such as *P. uterella* from the USA, *P. praecox* from Malta (MW305963), or the Brazilian *Phereoeca* sp. (MH540351.1), the latter of which was associated with *Rickettsia felis* (de [18,19]. These findings suggest the possibility of a distinct or previously undescribed species in Malaysia, underscoring the need for further taxonomic revision and molecular confirmation.

To investigate potential species divergence, phylogenetic comparisons were made with other Lepidoptera in the family Tineidae, including *Pelecystola* spp. from China [Yang and Li 2021] and *Monopis longella* from South Korea [20]. Additionally, comparisons included agriculturally relevant species such as *Mahasena corbetti* and *Metisa plana*, bagworms that cause economic damage in Malaysian oil palm plantations [21,22]. These broader comparisons help clarify genus-level relationships and highlight the genetic divergence of the Malaysian *Phereoeca* sp. from its closest relatives. The genetic distance observed among these species points toward potential cryptic speciation or geographic divergence in *Phereoeca* populations. Future studies using full mitochondrial genomes and morphological reassessment could help resolve these taxonomic ambiguities. Given the observed genetic divergence, formal taxonomic assessment in collaboration with tineid specialists will be necessary to determine the species status of the Malaysian population.

### Microbiome composition of *Phereoeca* sp.

This study provides the first comprehensive profile of the bacterial microbiome of *Phereoeca* sp., marking a significant contribution to understanding urban lepidopteran microbiota. The casebearer’s microbial community is shaped by its unique habitat which is the human household that differs from the microbiomes of agriculturally associated Lepidoptera such as *Metisa plana* and *Bombyx mori* [23,24]. Proteobacteria was the most dominant phylum in *Phereoeca* sp. (40.18%), followed by Actinobacteriota (32.13%) and Firmicutes (21.22%), a distribution that aligns with patterns found in other insects but with unique ratios. The prevalence of these phyla suggests they play critical roles in nutrient cycling, host physiology, and interactions within the urban environment [25]. Minor phyla such as Bacteroidota, Cyanobacteriota, and Actinomycetota were also detected and are notable for their potential involvement in histamine production and immune modulation [3].

At the family level, Pseudonocardiaceae, Beijerinckiaceae, and Burkholderiaceae were highly abundant, particularly within the Actinobacteriota and Proteobacteria phyla. These bacteria are typically associated with soil, plants, and environmental surfaces, suggesting potential transfer from household dust, textiles, or decaying organic matter. Enterobacteriaceae, though found in low abundance, remains a family of interest due to its relevance in both entomological and medical contexts, including gut symbiosis and pathogenicity [26]. Similar bacterial families have been recorded in lepidopterans such as *Galleria mellonella* and *Spodoptera frugiperda*, supporting the commonality of core gut bacteria across species. However, the differences in abundance and composition highlight how habitat and diet strongly influence microbial communities in urban versus agricultural insects.

The comparatively higher alpha diversity observed in the Negeri Sembilan samples may reflect local environmental or microhabitat differences influencing the microbial exposure of *Phereoeca* larvae. Variations in indoor humidity, household cleaning practices, building materials, or surrounding environmental conditions could contribute to differences in microbial acquisition, as environmental exposure is known to shape insect-associated microbial communities [24]. However, given the limited sample size and geographic coverage of the present study, these interpretations remain tentative. More systematic spatial sampling and environmental metadata collection will be necessary to determine the drivers of microbiome variation across locations.

It should be noted that the present microbiome profile primarily reflects bacteria associated with the processed larval material. Environmental or casing-associated microbes may also contribute to the detected community and should be examined separately in future studies.

### Histamine-associated bacteria and public health implications

A key focus of this study was the identification of histamine-associated bacteria, which could provide insights into anecdotal reports of skin irritation linked to *Phereoeca* larvae. Several genera detected such as *Enterobacter*, *Pseudomonas*, and *Streptomyces* are known histamine producers in both environmental and foodborne contexts [3]. For example, *Enterobacter hormaechei* produces histamine and can cause dermal toxicity, including severe lesions in mammals [27]. Ting et al. [24] also reported the presence of this bacterium in insect larvae, where it supports larval growth and digestion. Although *Enterobacter* spp. were not dominant in *Phereoeca* sp., their presence, even at low abundance, raises questions about their role in potential allergic reactions when the larvae interact with human environments.

The detection of *Mycobacterium tuberculosis* at a relative abundance of 2.46% was unexpected and warrants further attention. While it may result from environmental contamination, previous research has shown that ants and insects in hospital settings can act as vectors for *Mycobacterium* spp., including *M. tuberculosis* [28]. If *Phereoeca* sp. serves as a passive carrier, it could contribute to bacterial dispersion within households, although the direct transmission risk remains speculative. Furthermore, bacteria like *Pseudomonas fluorescens* and *Streptomyces griseus* were known histamine producers and were not found at the species level, but their genera were detected, indicating the potential presence of other histamine-active strains. These findings underscore the importance of future functional assays to confirm bacterial activity and their possible link to skin sensitivity or allergies in humans.

It is important to emphasize that the present study does not establish a direct causal relationship between the detected bacterial taxa and human dermatological or inflammatory outcomes. The microbiome data generated here should be interpreted as exploratory and hypothesis-generating. Many of the identified bacteria are known environmental or commensal taxa that are widely distributed across indoor surfaces and arthropods. Therefore, their detection in *Phereoeca* larvae does not by itself demonstrate species-specific medical relevance. Targeted functional assays, controlled exposure studies, and clinical correlation will be necessary to determine whether any of these microbial associates contribute meaningfully to reported skin irritation cases.

The absence of environmental surface controls or comparative household insect samples in the present study limits our ability to determine whether the observed microbiome profile is unique to *Phereoeca* sp. or reflects broader indoor microbial backgrounds. Future investigations incorporating parallel sampling of household surfaces and sympatric indoor insects would provide valuable context for interpreting host specificity.

In addition, the assignment of *M. tuberculosis* based on short-read 16S rRNA data should be interpreted with caution, as this marker region may not reliably discriminate among closely related *Mycobacterium* taxa. The present study did not assess epidemiological links to tuberculosis exposure, and confirmatory approaches (e.g., targeted PCR, culture-based methods, or longer-read sequencing) would be required to validate this identification.

### Possible histamine transmission via saliva or chewing activity

The idea that histamine or histamine-producing bacteria may be transmitted via insect oral secretions onto surfaces is plausible, especially considering larval feeding and movement patterns. Radwan-Oczko et al. [29] demonstrated the presence of histamine in saliva, particularly in autoimmune-related oral conditions, suggesting that oral bacteria can synthesize or trigger histamine release. If *Phereoeca* larvae deposit histamine on surfaces such as carpets, curtains, or clothing during feeding or pupation, human contact could lead to localized allergic reactions. Ribeiro [30] and Coutinho-Abreu et al. [31] have also shown that lepidopteran saliva can contain bioactive compounds with immunological effects. Additionally, Nässel [32] reported that histamine functions as a neurotransmitter in insects, indicating that it is physiologically active in their nervous systems and potentially present in body secretions.

This raises the hypothesis that bacterial symbionts in *Phereoeca* sp. may convert environmental or dietary histidine into histamine, which could accumulate on larval casing materials. Histamine production could be enhanced under certain environmental conditions, such as increased temperature or microbial load. As larvae often remain in fixed locations within households, repeated contact with contaminated casings may increase the likelihood of skin exposure. While this hypothesis is speculative, it aligns with the pattern of allergic symptoms reported online, though more rigorous clinical and biochemical testing is needed to establish causality. These insights provide a new direction for exploring insect-human interactions in urban ecosystems.

### Prevalent and unidentified microbial species

Among the top 25 species identified in this study, the two most abundant were *Methylobacterium-Methylorubrum* (11.79%) and *Burkholderia-Caballeronia-Paraburkholderia* (10.99%), both classified as unknown at the species level. These bacteria are typically associated with environmental sources such as soil, plants, and water systems, suggesting potential environmental acquisition by *Phereoeca* larvae. Their roles in histamine production remain unclear, but their high abundance may indicate ecological importance in the larval microbiome. Additionally, *Propionibacterium acnes* (now classified as *Cutibacterium acnes*) was found at 3.11% and is a known human skin commensal linked to acne. Its presence on *Phereoeca* larvae suggests possible contamination from human contact or shared living environments.

Other environmental bacteria, such as *Enhydrobacter aerosaccus* and *Mesorhizobium loti*, were also detected, although their relevance to human or insect physiology is limited. These species are more commonly associated with aquatic environments and plant nodules, respectively, and may reflect incidental microbial colonization. While their functional role in *Phereoeca* sp. remains uncertain, their detection adds to the complexity of the insect’s microbiome, which appears to integrate both environmental and host-derived bacteria. The presence of these species highlights the need for future studies using metatranscriptomics or metabolomics to better understand their biological significance. Overall, these findings lay the foundation for further exploration of microbial diversity and its implications for insect ecology and human health.

Barcoding and metagenomic analyses revealed that the household casebearer species studied, *Phereoeca* sp., may pose potential health risks to humans due to the presence of histamine-producing bacteria. These bacteria are likely capable of synthesizing bioactive compounds, such as histamine, which can trigger irritation and inflammatory responses upon contact with human skin. However, further investigation is essential to identify the specific bacterial strains involved, their enzymatic pathways, and the exact mechanisms through which these compounds exert physiological effects. Confirming the species identity of *Phereoeca* sp. will not only contribute to taxonomic clarity but also support the development of effective and environmentally safe household pest management strategies.

From an applied perspective, the present findings provide a preliminary basis for improving household pest awareness and management of casebearer infestations in indoor environments. Although direct health impacts remain to be experimentally validated, the detection of bacteria with potential dermatological relevance highlights the importance of routine household hygiene, monitoring of casebearer presence, and proper cleaning of wall and fabric surfaces where larvae commonly occur. Enhanced taxonomic resolution of Malaysian *Phereoeca* populations may also support more targeted pest management strategies in the future. At the public health level, these results primarily serve as an early evidence framework to guide further interdisciplinary investigations rather than to justify immediate intervention measures.

It is important to note that this study was based on a limited number of samples collected from selected locations in Peninsular Malaysia and therefore does not aim to represent the full geographic diversity of *Phereoeca* populations across Malaysia, including Malaysian Borneo. The findings should thus be interpreted as an initial baseline assessment. Future studies incorporating larger sample sizes and wider geographic coverage are needed to better resolve population-level patterns [33,34,35,36,37].

Despite its small size, this casebearer species may represent an under-recognized component of indoor arthropod communities, underscoring the need for deeper ecological, microbiological, and toxicological studies to better understand its role in the urban environment.

## Conclusions

This study presents the first combined molecular identification and microbiome survey of household casebearers collected from residential settings in Malaysia. COI barcoding indicates that the examined specimens form a distinct lineage within the genus *Phereoeca*, suggesting that the Malaysian population may represent an unresolved or potentially undescribed taxon. The 16S rRNA analysis revealed a diverse bacterial community dominated by Proteobacteria and Actinobacteriota, including several taxa that have previously been reported in contexts involving skin irritation or opportunistic infections. However, the present data do not demonstrate a direct causal link between *Phereoeca* larvae and human dermatological reactions, and many of the detected bacteria are commonly found in indoor environments and on other arthropods. The findings should therefore be viewed as preliminary. Further work, particularly functional assays, culture-based validation, and clinical correlation will be necessary to determine whether these insects contribute in any meaningful way to reported cases of household skin sensitivity. This study establishes a baseline for the taxonomy and microbial associations of *Phereoeca* in Malaysia and highlights the species as a still poorly studied member of indoor arthropod communities. Broader sampling and interdisciplinary follow-up studies will be important to better understand its ecological role and to clarify whether it has any verified relevance to indoor environmental health.

## Supporting information

S1 Fig Original uncropped and unadjusted gel image underlying Fig. 3a. (PDF)

S2 Fig Original uncropped and unadjusted gel image underlying Fig. 3b. (PDF)
