# Supplementary figures and images for: Molecular identification and microbiome profiling of household casebearer, Phereoeca sp. (Lepidoptera: Tineidae) from Malaysia: Potential implications for human skin irritation
Source: PLoS One. 2026 Apr 9;21(4):e0346590. doi: 10.1371/journal.pone.0346590 (PMC13065050; doi:10.1371/journal.pone.0346590)

S1 raw images (COI)

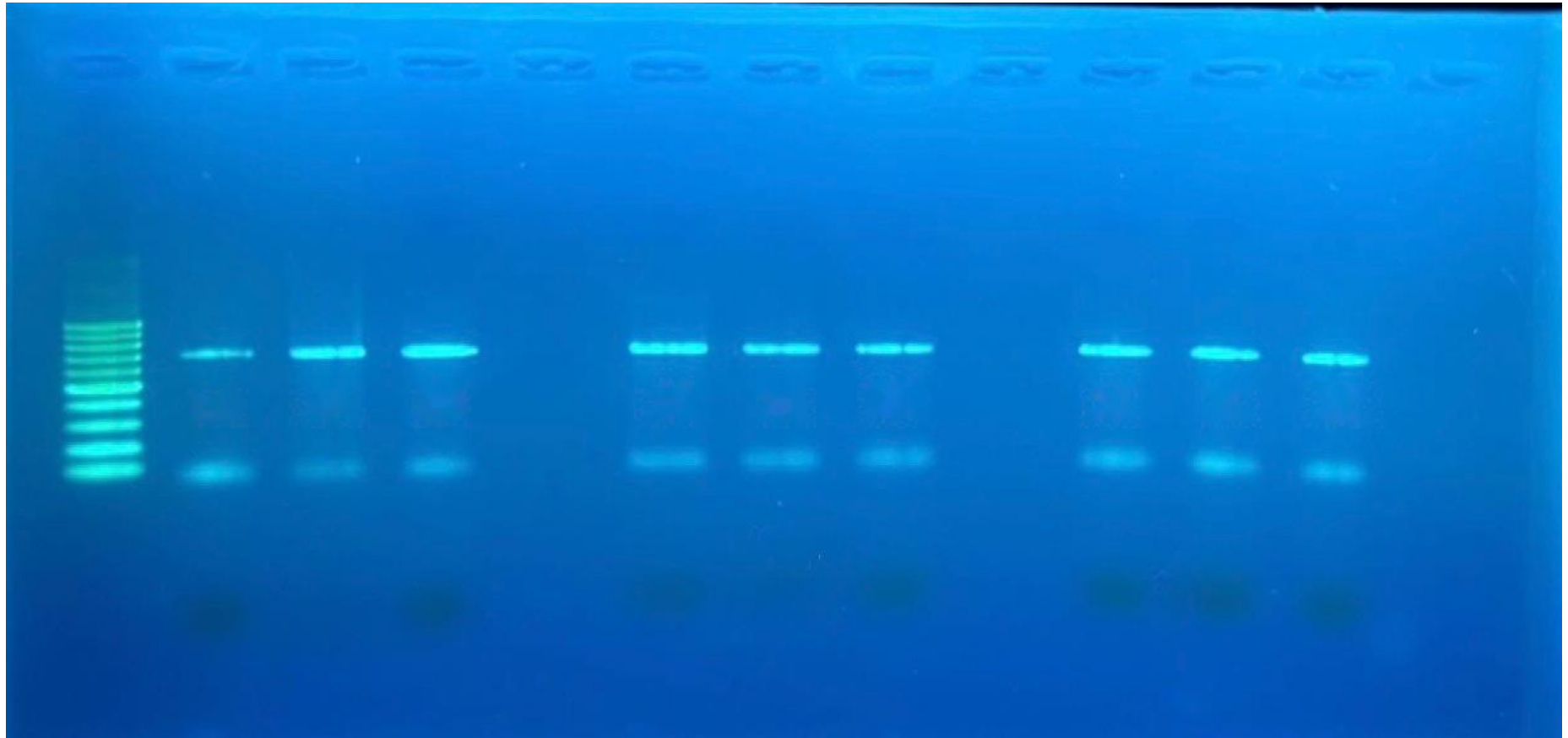

Supplement: S1 Fig — (PDF) [file pone.0346590.s001.pdf]

**S2 raw images (16S)**

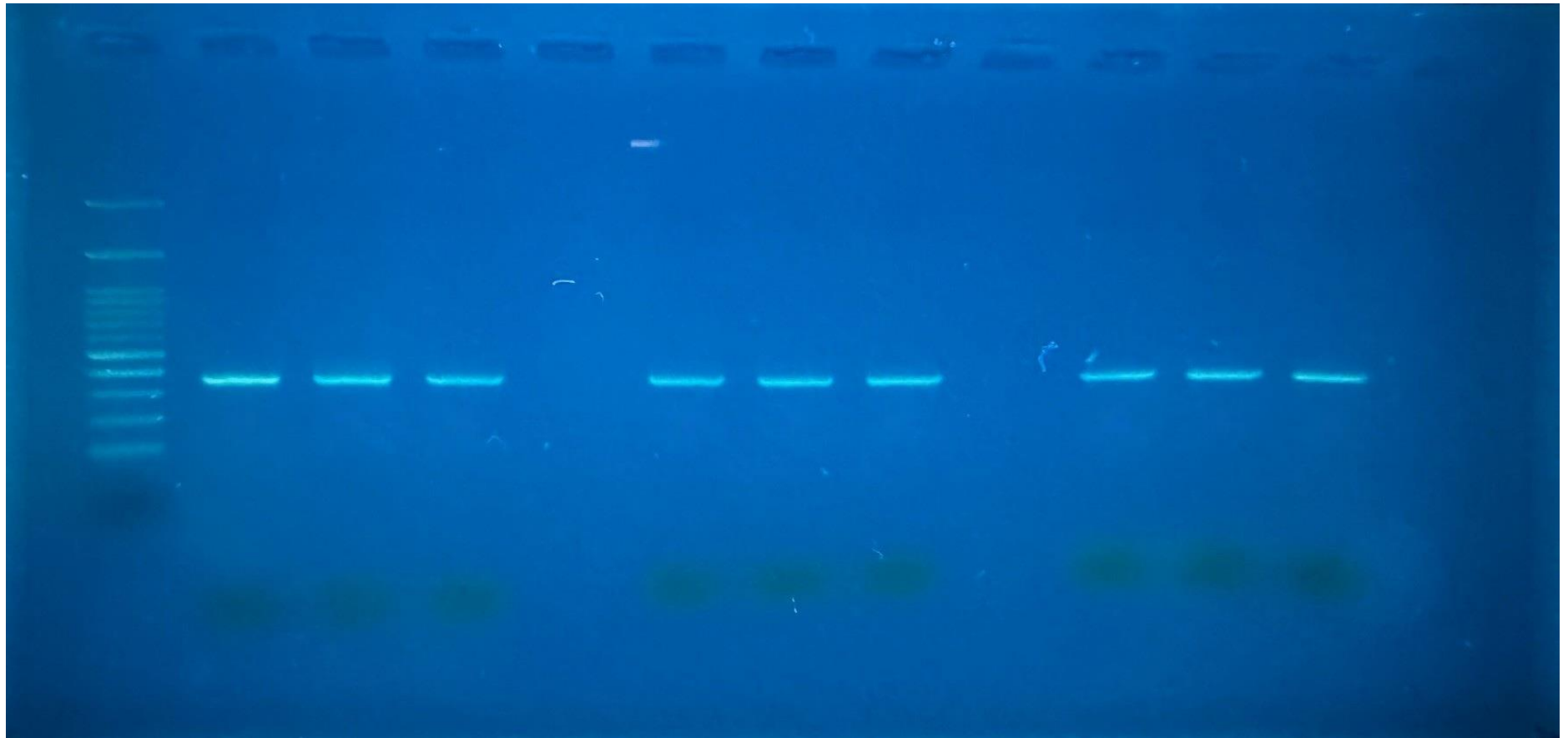

Supplement: S2 Fig — (PDF) [file pone.0346590.s002.pdf]
